# Supplementary material for: A systematic literature review on the applications of recurrent neural networks in code clone research
Source: PLoS One. 2024 Feb 2;19(2):e0296858. doi: 10.1371/journal.pone.0296858 (PMC10836701; doi:10.1371/journal.pone.0296858)
Supplement: S1 Appendix — (PDF) [file pone.0296858.s002.pdf]

List of Relevant studies

ID	Study Title
R1	*A code clone detection algorithm based on graph convolution network with AST tree edge*
R2	*A Novel Code Stylometry-based Code Clone Detection Strategy*
R3	*A novel neural source code representation based on abstract syntax tree*
R4	*Asteria: Deep Learning-based AST-Encoding for Cross-platform Binary Code Similarity Detection*
R5	*BinDeep: A deep learning approach to binary code similarity detection*
R6	*Clone-slicer: Detecting domain specific binary code clones through program slicing*
R7	*CodeGRU: Context-aware deep learning with gated recurrent unit for source code modeling*
R8	*CroLSSim: Cross-language software similarity detector using hybrid approach of LSA-based AST-MDrep features and CNN-LSTM model*
R9	*Cross-language clone detection by learning over abstract syntax trees*
R10	*Cross-Project Transfer Representation Learning for Vulnerable Function Discovery*
R11	*Deep code search*
R12	*Deep learning code fragments for code clone detection*
R13	*Deep Learning Similarities from Different Representations of Source Code*
R14	*DeepClone: Modeling Clones to Generate Code Predictions.*
R15	*FCCA: Hybrid Code Representation for Functional Clone Detection Using Attention Networks*
R16	*Focus: Function clone identification on cross-platform*
R17	*From Local to Global Semantic Clone Detection*
R18	*HELoC: hierarchical contrastive learning of source code representation*
R19	*Hierarchical Embedding for Code Search in Software Q&A Sites*
R20	*Large-scale and Robust Code Authorship Identification with Deep Feature Learning*
R21	*Learning-Based Recursive Aggregation of Abstract Syntax Trees for Code Clone Detection*
R22	*MulCode: A Multi-task Learning Approach for Source Code Understanding.*
R23	*Modular Tree Network for Source Code Representation Learning.*
R24	*Neural Detection of Semantic Code Clones Via Tree-Based Convolution*
R25	*Plagiarism Detection in Programming Assignments Using Deep Features.*
R26	*Plagiarism detection in students' programming assignments based on semantics: multimedia e-learning based smart assessment methodology*
R27	*Positive and unlabeled learning for detecting software functional clones with adversarial training*
R28	*SCDetector: software functional clone detection based on semantic tokens analysis*
R29	*SEED: Semantic Graph Based Deep Detection for Type-4 Clone*
R30	*Siamese-Based BiLSTM Network for Scratch Source Code Similarity Measuring*
R31	*Software code refactoring based on deep neural network-based fitness function*
R32	*Supervised deep features for software functional clone detection by exploiting lexical and syntactical information in source code*
R33	*Three-phase behavior-based detection and classification of known and unknown malware*
R34	*Towards Accurate Duplicate Bug Retrieval Using Deep Learning Techniques.*
R35	*Transformer-based networks over tree structures for code classification.* Applied Intelligence
R36	*Type4Py: practical deep similarity learning-based type inference for python*
R37	*Unified abstract syntax tree representation learning for cross-language program classification*
R38	*VDSimilar: Vulnerability detection based on code similarity of vulnerabilities and patches*
R39	*αDiff: cross-version binary code similarity detection with DNN*
R40	*Combining Holistic Source Code Representation with Siamese Neural Networks for Detecting Code Clones*
R41	*Deep learning application on code clone detection: A review of current knowledge*
R42	*Hierarchical semantic-aware neural code representation*
R43	*Improve Language Modeling for Code Completion Through Learning General Token Repetition of Source Code with Optimized Memory.*
R44	*Code Clone Detection with Hierarchical Attentive Graph Embedding*
R45	*Adaptive deep code search.*
R46	*Clone detection in 5Genabled social IoT system using graph semantics and deep learning model.*

R47	*FG-Droid: Grouping based feature size reduction for Android malware detection*
R48	*LSTM Hyper-Parameter Selection for Malware Detection: Interaction Effects and Hierarchical Selection Approach.*
R49	*Vulnerability Detection in Smart Contracts Using Deep Learning*.
R50	*Automated Software Vulnerability Detection via Pre-trained Context Encoder and Self Attention*
R51	*Bug localization with combination of deep learning and information retrieval*
R52	*Deep Convolutional and Recurrent Neural Networks for Cell Motility Discrimination and Prediction*
R53	*Deep learned BLSTM for online handwriting modeling simulating the Beta-Elliptic approach*

Quality Assessment Control Score

Study	QC1	QC2	QC3	QC4	QC5	QC6	QC7	QC8	QC9	QC10	QC11	QC12	Score
R1	0	1	0	0	0	0	0	0	0	0	0	0	0
R2	1	1	1	1	1	1	0	1	1	0	1	1	**10**
R3	1	1	1	1	1	1	1	1	1	1	1	1	**12**
R4	1	1	1	1	1	1	1	1	1	1	0.5	1	**11.5**
R5	1	1	1	1	1	1	1	0	1	0	0.5	1	**9.5**
R6	0	1	0	0	0	0	0	0	0	0	0	0	0
R7	1	0	0	0	0	0	0	0	0	0	0	0	0
R8	1	1	1	1	1	1	1	1	1	1	0.5	1	**11.5**
R9	1	1	1	1	1	1	1	1	1	1	0.5	1	**11.5**
R10	1	0	0	0	0	0	0	0	0	0	0	0	0
R11	1	0	0	0	0	0	0	0	0	0	0	0	0
R12	1	1	1	1	1	1	1	1	1	1	0.5	1	**11.5**
R13	1	1	1	1	1	1	1	0	1	1	1	1	**11**
R14	0	1	0	0	0	0	0	0	0	0	0	0	0
R15	1	1	1	1	1	1	1	1	1	1	1	1	**12**
R16	0	1	0	0	0	0	0	0	0	0	0	0	0
R17	1	1	1	1	1	1	0	1	1	1	0.5	1	**10.5**
R18	0	1	0	0	0	0	0	0	0	0	0	0	0
R19	1	0	0	0	0	0	0	0	0	0	0	0	0
20	1	0	0	0	0	0	0	0	0	0	0	0	0
R21	0	1	0	0	0	0	0	0	0	0	0	0	0
R22	1	1	1	1	1	1	1	1	1	1	0.5	1	**11.5**
R23	1	1	1	1	1	1	1	1	1	1	1	1	**12**
R24	0	1	0	0	0	0	0	0	0	0	0	0	0
R25	1	1	1	1	1	1	0	1	1	0	0.5	1	**9.5**
R26	0	1	0	0	0	0	0	0	0	0	0	0	0
R27	1	1	1	1	1	1	1	0	1	0	1	1	**10**
R28	1	1	1	1	1	1	1	1	1	1	1	1	**12**
R29	0	1	0	0	0	0	0	0	0	0	0	0	0
R30	1	1	1	1	1	1	1	1	1	0	0.5	1	**10.5**
R31	0	1	0	0	0	0	0	0	0	0	0	0	0
R32	1	1	1	1	1	1	0	1	1	0	1	1	**10**
R33	1	0	0	0	0	0	0	0	0	0	0	0	0
R34	1	0	0	0	0	0	0	0	0	0	0	0	0
R35	0	1	0	0	0	0	0	0	0	0	0	0	0
R36	1	0	0	0	0	0	0	0	0	0	0	0	0
R37	1	0	0	0	0	0	0	0	0	0	0	0	0
R38	1	1	1	1	1	1	1	1	1	1	0.5	1	**11.5**
R39	0	1	0	0	0	0	0	0	0	0	0	0	0
R40	0	1	0	0	0	0	0	0	0	0	0	0	0
R41	1	0	0	0	0	0	0	0	0	0	0	0	0
R42	1	1	1	1	1	1	1	1	1	1	0.5	1	**11.5**
R43	1	0	0	0	0	0	0	0	0	0	0	0	0
R44	0	1	0	0	0	0	0	0	0	0	0	0	0
R45	1	0	0	0	0	0	0	0	0	0	0	0	0
R46	1	1	1	1	1	1	1	1	1	0	0.5	1	**10.5**
R47	1	0	0	0	0	0	0	0	0	0	0	0	0
R48	1	0	0	0	0	0	0	0	0	0	0	0	0
R49	1	0	0	0	0	0	0	0	0	0	0	0	0
R50	1	0	0	0	0	0	0	0	0	0	0	0	0
R51	1	0	0	0	0	0	0	0	0	0	0	0	0
R52	1	0	0	0	0	0	0	0	0	0	0	0	0
R53	1	0	0	0	0	0	0	0	0	0	0	0	0
